# Football-Specific Performance and Cognitive Fatigue Resistance Following Supplementation with Two Phosphatidylserine Doses Combined with Taurine and Caffeine in Professional Male Football Players: A Randomized, Triple-Masked, Placebo-Controlled Trial

**DOI:** 10.3390/nu18162588

**Published:** 2026-08-07

**Authors:** Krzysztof Mizera, Dominik Mazurek, Alejandro Martínez-Rodríguez, Anna Kęska

**Affiliations:** 1 School of Medical & Health Sciences, VIZJA University in Warsaw, 01-143 Warsaw, Poland; 2 Śląsk Wrocław Football Club, 54-118 Wrocław, Poland; 3 Department of Analytical Chemistry, Nutrition and Food Sciences, Faculty of Sciences, University of Alicante, 00-968 Warszawa, Spain; 4 Department of Biochemistry and Biology, Józef Piłsudski University of Physical Education in Warsaw, 00-968 Warsaw, Poland

**Keywords:** ergogenic aids, sports nutrition, team sports, mental fatigue, reaction time, psychomotor function, high-intensity running, external load, GPS monitoring

## Abstract

Background/Objectives: Cognitive fatigue and impaired psychomotor function may contribute to reductions in football-specific performance during prolonged match-like activity. This study evaluated cognitive, psychomotor, sprint, and football-specific locomotor responses to multi-ingredient protocols containing taurine, caffeine, and either 300 or 600 mg·day^−1^ of phosphatidylserine in professional male football players. Methods: In this randomized, triple-masked, placebo-controlled, parallel-group trial, 97 professional male football players were allocated to placebo (PLA, *n* = 24), taurine plus caffeine (TC, *n* = 24), taurine plus caffeine plus phosphatidylserine 300 mg·day^−1^ (TCP-300, *n* = 24), or taurine plus caffeine plus phosphatidylserine 600 mg·day^−1^ (TCP-600, *n* = 25) for 14 days. Taurine and caffeine doses were 1500 mg·day^−1^ and 200 mg·day^−1^, respectively. Outcomes included 30 m sprint performance, visual and auditory reaction time, Stroop-task performance, perceived mental fatigue, GPS-derived locomotor variables, and post-intervention first-to-second-half performance-loss indices during a standardized football-specific exercise protocol. Results: The baseline-adjusted ANCOVA demonstrated a significant treatment-group effect on post-intervention 30 m sprint time. After Holm–Bonferroni correction, significant treatment-related effects remained for Stroop reaction time, visual reaction time, and perceived mental fatigue. No statistically significant treatment-related effect was observed for auditory reaction time (unadjusted *p* = 0.074; Holm-adjusted *p* = 0.096). During the post-intervention standardized football-specific protocol, baseline-adjusted GPS-derived locomotor outcomes differed significantly between groups. First-to-second-half performance-loss indices also differed between groups, with the smallest post-intervention losses generally observed in TCP-600. Because comparable decline indices were unavailable at baseline, these findings do not represent intervention-related changes in fatigue resistance. Conclusions: Under the present 14-day experimental conditions, the multi-ingredient protocols containing taurine, caffeine, and phosphatidylserine were associated with more favorable selected cognitive and psychomotor outcomes, 30 m sprint performance, and baseline-adjusted GPS-derived locomotor outcomes than placebo. The groups also differed in post-intervention first-to-second-half performance loss during the standardized football-specific protocol. Because corresponding decline indices were not assessed at baseline, these findings do not demonstrate an intervention-related improvement in fatigue resistance. Numerically more favorable values were observed for several outcomes in TCP-600; however, differences between TCP-300 and TCP-600 were modest and not consistently statistically significant, and the study was not specifically powered to establish a dose–response relationship. Because phosphatidylserine was not tested independently, these findings should be interpreted as responses to the combined multi-ingredient supplementation protocols rather than as effects of phosphatidylserine alone.

## 1. Introduction

Football is a high-intensity intermittent sport in which players repeatedly sprint, change direction, and make technical and tactical decisions while fatigue is gradually increasing. In practice, successful performance does not depend on one isolated ability. Players have to preserve physical output, cognitive efficiency, technical quality, and tactical discipline for most of the match, including the periods in which fatigue is already clearly present. Match and seasonal performance are influenced by neuromuscular, endurance-related, and game-related factors that fluctuate across the competitive season and contribute to changes in football-specific performance outcomes [1]. Previous studies in elite soccer players have shown marked reductions in high-intensity running and repeated-sprint capacity in the later stages of match play, which makes fatigue-related mechanisms particularly relevant when analysing football-specific performance decline [2].

During match play, players typically cover 10–13 km, but this distance alone does not fully reflect the demands of the game. Within this distance, players perform repeated high-intensity actions, react to rapidly changing situations, and make frequent decisions under pressure [2]. Match play is also associated with higher energy expenditure and a greater proportion of vigorous and very vigorous activities than training sessions, confirming that competitive football imposes substantial physiological stress [3]. However, physical capacity is only part of the performance profile. Executive functions have also been identified as predictors of success in elite soccer players, which supports the view that cognitive performance is an important component of football-specific performance [4]. Experimental evidence indicates that mental fatigue can impair GPS-derived physical activity, technical execution, and decision-making performance during soccer-specific intermittent exercise [5]. Evidence is also accumulating that the ability to resist cognitive fatigue may be important for maintaining performance during prolonged exercise [6].

Previous football research has examined caffeine-containing supplementation, dietary habits, body composition, physiological responses, and supplement-use practices in players [7,8,9], underscoring the applied relevance of nutritional strategies in this population. In recent years, more attention has been paid to nutritional strategies that could support several domains at once: physical performance, resistance to fatigue, and cognitive functioning during intermittent exercise. A systematic review in elite soccer players concluded that dietary supplements may influence selected sport-specific performance outcomes, although the size of these effects depends on the supplement, dosage, timing, and the type of exercise used in the study [10]. Similarly, a systematic review and meta-analysis of creatine supplementation suggested potential benefits for repeated-sprint performance and training adaptations in soccer players, despite considerable differences between study protocols and doses [11]. Recent network meta-analytic evidence also indicates that the effectiveness of a supplement may depend on the specific performance domain being assessed [12].

Caffeine is still one of the best-studied ergogenic aids in sport nutrition. Current evidence suggests that caffeine may improve physical performance, vigilance, attention, and reaction time, especially when exercise is performed under fatigue [13]. A systematic review focused on soccer players reported improvements in selected physical performance outcomes and reductions in perceived fatigue during soccer-related exercise [14]. These effects are usually explained mainly by adenosine receptor antagonism, which increases central nervous system activation and modifies neurotransmission [13].

Taurine has also become increasingly interesting in sports nutrition, although the evidence is not as extensive as for caffeine. This sulfur-containing amino acid is involved in osmoregulation, calcium handling, membrane stabilization, and neuromuscular function [15]. Studies by De Carvalho et al. suggested that taurine supplementation may support exercise performance and reduce fatigue-related impairments, probably through mechanisms linked to excitation–contraction coupling, antioxidant defense, and skeletal muscle function [16].

Phosphatidylserine (PS) is another compound relevant in this context because of its possible effects on cognitive function, stress regulation, and exercise tolerance. As an important phospholipid component of cell membranes, PS is involved in neurotransmission and central nervous system activity [17]. Human trials have reported improvements in a sport-specific performance task in golfers [18] and attenuated adrenocorticotropic hormone and cortisol responses to exercise-induced physical stress [19]. However, not all findings are positive. Other studies did not show clear effects on oxidative stress, cortisol responses, muscle soreness, or markers of muscle damage after strenuous exercise [20]. Therefore, phosphatidylserine remains a candidate component of multi-ingredient strategies, but evidence regarding its independent effects in sport-specific settings is limited and inconsistent.

Central fatigue mechanisms, including neurotransmitter regulation and activation of the hypothalamic–pituitary–adrenal axis, may contribute to reductions in cognitive and physical performance during prolonged exercise [21]. Nutritional interventions that influence these processes may therefore be relevant for football-specific performance, particularly when cognitive and physical fatigue occur together.

Although caffeine, taurine, and phosphatidylserine have each been investigated in relation to exercise performance or cognitive function, evidence regarding multi-ingredient protocols combining these compounds under standardized football-specific conditions remains limited. In particular, it is unclear whether phosphatidylserine-containing protocols administered on a fixed taurine–caffeine background are associated with different performance responses and whether the 300 and 600 mg·day^−1^ conditions show distinct numerical patterns. This question is relevant in football, where players must maintain sprint ability, psychomotor performance, decision-making, and high-intensity locomotor output while physical and cognitive fatigue develop concurrently. Mizera et al. [22] previously evaluated a related taurine-, caffeine-, and phosphatidylserine-containing supplementation protocol in professional football players. The present investigation was conducted as a completely independent trial, with no overlap in participants, clubs, intervention periods, or individual-level data between the studies. It extends the earlier work through a 14-day, triple-masked, four-group pre–post design comparing placebo, taurine plus caffeine, and taurine plus caffeine combined with either 300 or 600 mg·day^−1^ of phosphatidylserine, together with expanded assessment of cognitive-load responses, sprint performance, and GPS-derived outcomes. Therefore, the aim of the present study was to evaluate whether adding 300 or 600 mg·day^−1^ of phosphatidylserine to taurine and caffeine supplementation influenced cognitive, psychomotor, sprint, locomotor, and fatigue-related outcomes. We hypothesized that the phosphatidylserine-containing multi-ingredient protocols would produce more favorable changes in the prospectively registered primary outcome, 30 m sprint time, than placebo and taurine plus caffeine. For the revised manuscript, cognitive, psychomotor, locomotor, and fatigue-related outcomes were interpreted as supportive secondary outcomes, whereas technical and tactical outcomes were treated as exploratory. This interpretive hierarchy was formalized during revision and was not part of a prospectively documented statistical analysis plan. Differences between the TCP-300 and TCP-600 conditions were interpreted exploratorily rather than as a confirmatory dose–response comparison.

## 2. Materials and Methods

### 2.1. Study Design and Participants

The study was conducted in accordance with the Declaration of Helsinki. All participants provided written informed consent before participation. The protocol was approved by the Ethics Committee of the University of Economics and Human Sciences in Warsaw (approval number: 01/11/2024; approved on 12 November 2024) and prospectively registered at ClinicalTrials.gov (NCT07604519). The 30 m sprint time was prospectively registered as the primary outcome. Neither a separate full study protocol nor a standalone statistical analysis plan was finalized or deposited in a public repository. The baseline-adjusted ANCOVA model, the five multiplicity families, and the primary/supportive/exploratory interpretive hierarchy were formalized during manuscript revision, after group unblinding and examination of the initial outcome results. These revision-stage analytical decisions were therefore not prospectively prespecified.

Athletes and members of the public were not involved in the design, conduct, analysis, interpretation, or reporting of the study beyond their participation as study participants.

A randomized, triple-masked, placebo-controlled, parallel-group, pre–post intervention design was used. The study was reported in accordance with the CONSORT guidelines for randomized controlled trials. Details regarding randomization, allocation concealment, masking, and compliance monitoring are provided below. Ninety-seven highly trained male football players from three professional clubs participated in the study. The sample comprised first-team players together with selected reserve-team and U19 players who were regularly involved in first-team training. All participants, including those from the reserve-team and U19 squads, held professional player contracts with their respective clubs during the study period. Accordingly, the term “professional male football players” refers to contractual status rather than exclusively to first-team squad membership. All participants trained within professional club environments and were exposed to comparable club-based training loads. Testing was conducted during the pre-season period under comparable environmental conditions and at similar times of day. Following randomization, participants were assigned to placebo (PLA, *n* = 24), taurine + caffeine (TC, *n* = 24), taurine + caffeine + phosphatidylserine 300 mg/day (TCP-300, *n* = 24), or taurine + caffeine + phosphatidylserine 600 mg/day (TCP-600, *n* = 25).

Participants were 18–35 years old (24.8 ± 3.9 years), with a body mass of 79.0 ± 3.3 kg, height of 179 ± 4 cm, and body fat percentage of 12.5 ± 2.0%. Detailed characteristics are presented in Table 1. Body composition was assessed using a bioelectrical impedance analyzer (InBody 180, Biospace Co., Seoul, Republic of Korea).

Players trained approximately 1.5–2 h·day^−1^, 6 days·week^−1^, and continued regular team training and match-play activities throughout the pre-season period.

Inclusion criteria were regular participation in professional football training, at least five years of structured football training experience, and absence of injury during testing. Exclusion criteria included current injury, chronic disease, regular medication use, smoking, caffeine hypersensitivity, regular use of ergogenic supplements, or inability to complete the study protocol.

Participants refrained from caffeine-containing products and dietary supplements for seven days before baseline testing. Participants continued to avoid all non-study caffeine-containing products throughout the intervention period, such that caffeine exposure in the active conditions was derived exclusively from the assigned study supplementation. Habitual caffeine intake was assessed using a standardized questionnaire. Participants otherwise maintained their usual diet throughout the intervention. Dietary intake was monitored using 3-day dietary records collected before baseline and post-intervention testing. Training load during the 72 h preceding testing was standardized and monitored using session-RPE and team training documentation.

### 2.2. Experimental Design and Supplementation

Participants were randomly assigned to one of four experimental groups using stratified randomization. Supplements and placebo capsules were identical in appearance, taste, color, and packaging to maintain masking throughout the masked phase of the trial.

The experimental conditions were as follows:

PLA: Placebo capsules;

TC: Taurine (1500 mg/day) + caffeine (200 mg/day);

TCP-300: Taurine (1500 mg/day) + caffeine (200 mg/day) + phosphatidylserine (300 mg/day);

TCP-600: Taurine (1500 mg/day) + caffeine (200 mg/day) + phosphatidylserine (600 mg/day).

Taurine was supplied as 500 mg capsules (NOW Taurine 500 mg, NOW Foods, Bloomingdale, IL, USA), caffeine was provided as an anhydrous caffeine product containing 200 mg per tablet (GymBeam Caffeine, GymBeam s.r.o., Košice, Slovakia), and phosphatidylserine was provided as 150 mg capsules (Probase Nutrition, Calabasas, CA, USA). The products were purchased through distributors operating in Poland. Product identity and nominal active-ingredient content were verified against the manufacturers’ labels and product specifications; no additional independent laboratory analysis was performed.

To maintain masking, the commercial products were over-encapsulated in identical opaque study capsules, with microcrystalline cellulose used as the inert filler. Each participant received eight study capsules per day: eight placebo capsules in PLA; three taurine capsules, one caffeine-containing capsule, and four placebo capsules in TC; three taurine capsules, one caffeine-containing capsule, two phosphatidylserine capsules, and two placebo capsules in TCP-300; and three taurine capsules, one caffeine-containing capsule, and four phosphatidylserine capsules in TCP-600. Microcrystalline cellulose was added where necessary to standardize capsule number, appearance, and total mass across conditions.

The supplementation period lasted 14 consecutive days. The complete daily dose was administered once daily, approximately 60 min before the scheduled training session and approximately 2 h after the preceding meal. On the final testing day, participants reported to the club, consumed the assigned supplementation under supervision, and then completed the routine pre-testing procedures, including the team briefing and preparation period. The first assessment began approximately 45–60 min after ingestion, after which the cognitive, sprint, and football-specific assessments were performed in the standardized order described below. On non-training days, the complete dose was administered at approximately the same time of day as the participant’s habitual training-time dose and approximately 2 h after the preceding meal.

Caffeine dosage was standardized across all active supplementation conditions. Based on mean baseline body mass, the fixed 200 mg caffeine dose corresponded to approximately 2.53 mg·kg^−1^ in TC, 2.54 mg·kg^−1^ in TCP-300, and 2.53 mg·kg^−1^ in TCP-600.

### 2.3. Randomization, Masking, and Compliance Monitoring

Randomization was performed by an independent researcher using a computer-generated sequence with concealed block sizes. Stratification was based on baseline 30 m sprint time to support balance in the prospectively registered primary outcome.

The trial was registered as triple-masked. Participants, supplementation supervisors, and the investigators responsible for testing and outcome assessment remained unaware of treatment allocation throughout data collection. The same investigators performed both study testing and outcome assessment; these were not separate personnel. The two coaches who independently assessed the technical and tactical video recordings were also masked to treatment-group allocation and supplementation condition.

The investigators responsible for the initial statistical analyses used coded group labels and remained unaware of group identities until completion of those analyses. The allocation codes were then released. The baseline-adjusted ANCOVA and multiplicity framework introduced during manuscript revision were developed and applied after group unmasking.

The independent researcher who generated the randomization sequence and the independent club representative responsible for maintaining the allocation codes had access to the allocation information but had no role in recruitment, supplementation supervision, testing, outcome assessment, or statistical analysis. Masking effectiveness was not formally assessed.

Every scheduled intake was directly supervised by a masked member of the club’s performance staff, either a strength and conditioning coach or physiotherapist, and documented immediately in a daily compliance log. Participants consumed the complete daily dose in the presence of the supervising staff member. Adherence was additionally verified by counting any returned unused capsules at the end of the intervention.

Participants were asked daily about potential adverse effects, including gastrointestinal symptoms, sleep disturbances, headache, palpitations, nervousness, or other unusual symptoms, and no adverse events or protocol deviations were reported.

### 2.4. Baseline and Post-Intervention Testing Design

Before supplementation, participants completed baseline testing including:

- Sprint performance of 30 m;
- Visual reaction time;
- Auditory reaction time;
- Perceived mental fatigue;
- Cognitive fatigue protocol based on a modified computerized Stroop task.

During the familiarization session conducted before supplementation, participants also completed the football-specific exercise protocol with GPS monitoring to establish baseline locomotor performance.

At post-intervention, assessments were completed in the following standardized order: pre-task perceived-mental-fatigue assessment, 15 min modified Stroop task, immediate post-task perceived-mental-fatigue assessment, visual reaction-time test, auditory reaction-time test, standardized field warm-up, three 30 m sprint trials, and the 90 min football-specific exercise protocol. The same relative order of the cognitive, psychomotor, warm-up, and sprint assessments was maintained at baseline. Baseline GPS-derived data were collected during the separate familiarization football-specific session described above, using the same football-specific protocol and GPS procedures as at post-intervention.

### 2.5. Cognitive Fatigue Protocol

At baseline and post-intervention, participants completed a 15 min modified computerized Stroop task used as a brief standardized cognitive-load challenge. A 15 min Stroop condition has previously been applied in professional male soccer players, although longer protocols have generally produced clearer mental-fatigue-related performance impairments. The shorter duration was selected to balance cognitive demand with feasibility within professional football schedules. The task lasted 15 min and consisted of standardized congruent and incongruent trials presented in a fixed proportion. Stimuli were displayed on identical devices in a quiet indoor environment under standardized lighting and seating conditions. Participants identified the color of the stimuli as quickly and accurately as possible, and reaction time, accuracy, and error rate were recorded automatically. Before baseline assessment, all participants completed a familiarization session to reduce learning effects. Mean reaction time was calculated from valid responses only, and trials with anticipatory or implausibly delayed responses were excluded according to predefined criteria. Perceived mental fatigue was assessed immediately before and after the task using a 0–10 visual analogue scale (VAS). Perceived mental fatigue was analyzed using ratings obtained immediately before and after the Stroop task at both baseline and post-intervention. Task-induced change scores were calculated as post-task minus pre-task values. At post-intervention testing, the same testing setup, instructions, and transition times were maintained before participants proceeded to the standardized warm-up and football-specific exercise protocol.

### 2.6. Football-Specific Exercise Protocol

Following completion of the cognitive-load and psychomotor assessments described above, participants proceeded to the field and completed a standardized 15 min warm-up, the 30 m sprint trials, and the 90 min football-specific exercise protocol. The football-specific protocol was designed to reproduce selected physiological, locomotor, and cognitive demands relevant to football while maintaining experimental standardization. The protocol was developed in collaboration with the coaching staff and consisted of the following standardized components:

- 15 min technical drills;
- 30 min match-simulation play;
- 15 min football-specific drills;
- 30 min match-simulation play.

The 90 min football-specific phase was divided into two 45 min periods separated by a standardized 3–4 min recovery interval. All sessions were performed on natural grass under comparable environmental conditions and at similar times of day.

Locomotor activity and external load were continuously monitored during the football-specific phase using wearable GPS devices (Catapult Sports, Melbourne, Australia; 10 Hz GPS, 100 Hz triaxial accelerometer) positioned between the scapulae in a manufacturer-provided harness. Analyses were restricted to the 90 min football-specific phase and excluded the warm-up and cognitive fatigue protocol.

Training load during the 72 h preceding testing was standardized and monitored using session-RPE and team training documentation. Internal load during the football-specific protocol was assessed using the Borg CR10 scale 15–20 min after session completion.

### 2.7. Performance Monitoring

During the football-specific protocol, locomotor activity and external load were assessed using GPS-derived variables.

The analyzed variables included total distance covered, relative distance covered (m·min^−1^), high-speed running distance, number of sprints, maximum speed, and PlayerLoad™. High-speed running was defined as >19.8 km·h^−1^, whereas sprinting was defined as >25.0 km·h^−1^.

Within-session performance-loss variables were calculated by comparing the first and second halves of the football-specific protocol. These variables included sprint decline, maximum-speed decline, high-speed-running decline, and PlayerLoad decline. For each participant, percentage change was calculated as [(second-half value − first-half value)/first-half value] × 100, after which group means and standard deviations were calculated.

These within-session performance-loss indices were assessed only during the post-intervention football-specific protocol. Accordingly, these variables represented within-session changes from the first to the second half of the same post-intervention session and were not interpreted as pre–post changes or improvements.

The session was additionally recorded using drone video footage. Technical and tactical outcomes were treated as exploratory and were independently assessed from the video recordings by two football coaches using standardized scoring sheets and predefined observational criteria. Passing accuracy was calculated as the percentage of successfully completed passes relative to all attempted passes. Ball recoveries were defined as instances in which a player regained controlled possession following an opponent’s action. Technical errors were defined as unsuccessful technical actions resulting in loss of possession or failure to complete the intended action. Decision-making performance was rated independently by each coach on a 1–10 scale, where 1 indicated a clearly inappropriate tactical decision, and 10 indicated the optimal decision given the available tactical options; higher scores represented better decision-making performance.

Both video raters were masked to treatment-group allocation and supplementation condition. Before formal scoring, the raters reviewed the operational definitions and scoring criteria to ensure consistent interpretation of each outcome. Both raters independently assessed all recordings, without consultation during the initial scoring process. Inter-rater reliability was calculated separately for passing accuracy, decision-making score, ball recoveries, and technical errors from the initial independent ratings before any discussion between raters. Discrepant ratings were subsequently reviewed jointly, and a consensus score was used in the final statistical analyses. Inter-rater reliability was evaluated using a two-way mixed-effects, absolute-agreement, single-measures intraclass correlation coefficient model, with ICC estimates reported together with 95% confidence intervals.

### 2.8. Psychomotor and Sprint Testing

The predefined primary outcome was 30 m sprint time. Sprint performance was assessed using electronic timing gates. Participants performed three maximal 30 m sprint trials separated by standardized recovery periods, and the best trial was used for analysis.

Reaction time was assessed using two complementary methods. Visual reaction time was measured using a YB-1A reaction timer, whereas auditory reaction time was assessed using a computerized test implemented in PsychoPy software (version 2024.1.5) with randomized interstimulus intervals (2–5 s). Following familiarization, 10 valid trials were included in the final analysis, and the mean value was used for statistical evaluation. Valid trials were defined as responses recorded within the predefined acceptable response window, excluding anticipatory responses and technical errors. Trial order, instructions, and rest intervals were standardized across participants and time points. All devices were calibrated before testing, and assessments were conducted in a quiet room under controlled environmental conditions.

### 2.9. Outcome Prioritization

The 30 m sprint time was prospectively registered as the primary outcome. Cognitive fatigue resistance was operationalized as the maintenance or more favorable change in performance during the standardized Stroop-based cognitive-load challenge. It was assessed using Stroop reaction time, accuracy, error rate, and the task-induced change in perceived mental fatigue. No composite cognitive-fatigue-resistance score was calculated. Visual and auditory reaction times were treated as complementary psychomotor outcomes rather than direct indicators of cognitive fatigue resistance.

For the revised manuscript, cognitive, psychomotor, locomotor, and fatigue-related outcomes were organized as supportive secondary outcomes, whereas technical, tactical, and coach-rated observational variables were treated as exploratory and hypothesis-generating. This interpretive hierarchy was formalized during revision and was not prospectively prespecified. It was introduced to distinguish the registered primary outcome from the broader supportive and exploratory outcome set and to reduce overinterpretation. Because no formal a priori sample-size calculation or prospectively finalized statistical analysis plan was available, the study was not considered a fully confirmatory efficacy trial.

The supportive secondary outcome set comprised visual reaction time, auditory reaction time, perceived mental fatigue, Stroop task reaction time, Stroop task accuracy, Stroop task error rate, total distance covered, relative distance covered, sprint count, sprint decline, maximum speed, maximum speed decline, high-speed running distance, high-speed running decline, PlayerLoad, and PlayerLoad decline.

Exploratory outcomes included technical, tactical, and coach-rated observational variables. The ClinicalTrials.gov record also included heart-rate variability, sleep duration, sleep efficiency, subjective fatigue, and muscle soreness as recovery-related secondary outcomes. Data collection for all five outcomes was completed as part of the same trial. These outcomes fall outside the scientific scope of the present performance-focused manuscript. Data verification, completeness checks, and outcome-specific analyses are ongoing and have not yet been finalized. The findings have not been published and are intended for exclusive reporting in a separate companion manuscript focused on autonomic recovery, sleep, perceived recovery, and training readiness. Their exclusion from the present report was based on the distinct scientific scope of the outcome domains and not on the direction or statistical significance of the findings. To prevent inappropriate overlap, the companion manuscript will not reproduce the 30 m sprint, cognitive, psychomotor, GPS-derived locomotor, within-session decline, or technical and tactical outcomes reported in the present article.

### 2.10. Statistical Analysis

All statistical analyses were performed using IBM SPSS Statistics for Windows (Version 29.0; IBM Corp., Armonk, NY, USA).

Participant recruitment was based on the availability of eligible professional football players during the study period. Therefore, a convenience sample was used. A total of 97 participants were enrolled and randomized across four groups. No formal a priori sample size calculation was performed, as recruitment depended on the availability of professional football players during the pre-season period. Because no formal a priori sample size calculation was performed, statistical power was not prospectively established for the secondary or exploratory outcomes. Consequently, findings beyond the predefined primary endpoint were interpreted as supportive or preliminary, whereas technical and tactical outcomes were regarded as exploratory and hypothesis-generating rather than confirmatory. To limit overinterpretation, the primary, secondary, and exploratory outcomes were classified separately, effect sizes with confidence intervals were reported, and multiplicity was addressed using the family-level and pairwise Holm–Bonferroni procedures described below.

Normality was assessed using the Shapiro–Wilk test and visual inspection of Q–Q plots. Baseline differences between groups were evaluated using one-way analysis of variance (ANOVA) or the Kruskal–Wallis test, as appropriate. Categorical participant characteristics were reported as counts and within-group percentages. Differences in the distributions of club affiliation, squad status, and primary playing position across randomized groups were assessed using Pearson’s chi-square test or the Fisher–Freeman–Halton exact test with Monte Carlo estimation when expected cell counts were sparse.

Visual reaction time, auditory reaction time, Stroop reaction time, Stroop accuracy, and Stroop error count were analyzed using mixed-model repeated-measures ANOVA, with treatment group as the between-subject factor and intervention time (baseline and post-intervention) as the within-subject factor. The group × intervention time interaction was used as the treatment-effect test for these outcomes.

Perceived mental fatigue was analyzed using a mixed-model repeated-measures ANOVA with treatment group as the between-subject factor and intervention time (baseline and post-intervention) and task phase (pre- and post-Stroop) as within-subject factors. The group × intervention time × task phase interaction was used to determine whether supplementation modified the task-induced fatigue response. Task-induced change scores were calculated as post-task minus pre-task values.

The mixed-model repeated-measures ANOVA was the principal analysis of 30 m sprint performance used in the original manuscript. During revision, a baseline-adjusted ANCOVA was adopted as the main analysis of the prospectively registered 30 m sprint outcome, with post-intervention sprint time as the dependent variable, treatment group as the fixed factor, and baseline sprint time as the covariate. This decision was made after group unblinding and examination of the initial outcome results and was therefore not prospectively prespecified. Estimated marginal means, adjusted between-group differences, 95% confidence intervals, and partial eta-squared were reported. Pairwise comparisons were adjusted using the Holm–Bonferroni procedure. The repeated-measures group × intervention-time analysis was retained as a supportive sensitivity analysis providing a complementary assessment of change over time. Each GPS-derived outcome was analyzed using a separate analysis of covariance model. The post-intervention value was entered as the dependent variable, treatment group as the fixed factor, and the corresponding baseline value of the analyzed outcome as the covariate. Accordingly, separate models were fitted for total distance, relative distance, high-speed running distance, sprint count, maximum speed, and PlayerLoad. Baseline-adjusted post-intervention estimated marginal means and adjusted between-group differences with 95% confidence intervals were obtained from each model. The linearity of the relationship between baseline and post-intervention values, homogeneity of regression slopes, residual normality, and homoscedasticity were evaluated. In sensitivity analyses, club affiliation was additionally included as a fixed factor.

Because corresponding baseline decline indices were unavailable, these variables were analyzed as supportive post-intervention between-group outcomes. Baseline 30 m sprint time, which was used as the randomization stratification variable and represented baseline sprint ability, was included as an adjustment covariate. These analyses assessed differences between groups in the magnitude of within-session decline and did not estimate pre–post treatment effects. Post hoc exploratory linear trend analyses were conducted across the TC, TCP-300, and TCP-600 conditions. These analyses were not prespecified, were interpreted as supportive only, and were not used independently to establish a dose–response relationship.

Exploratory technical and tactical outcomes were analyzed using one-way ANOVA. Pairwise comparisons were considered only for outcomes that remained statistically significant after family-level multiplicity correction.

During manuscript revision, the 21 outcome-level treatment-effect tests were organized into five conceptually defined families: the prospectively registered primary outcome (30 m sprint time analyzed using baseline-adjusted ANCOVA; *n* = 1), cognitive and psychomotor outcomes (*n* = 6), GPS-derived locomotor outcomes (*n* = 6), within-session performance-loss outcomes (*n* = 4), and exploratory technical and tactical outcomes (*n* = 4). For perceived mental fatigue, the group × intervention time × task phase interaction was treated as the single treatment-effect test. This family structure and the associated multiplicity framework were not prospectively prespecified; they were introduced during revision, after examination of the initial results, to provide a more transparent and conservative organization of the outcome set. No multiplicity adjustment was applied to the single prospectively registered primary outcome. Within each supportive secondary or exploratory family, omnibus *p*-values were adjusted using the Holm–Bonferroni step-down procedure. Pairwise comparisons were undertaken only for outcomes that remained significant after family-level correction, and the six comparisons among the four groups were then Holm–Bonferroni-adjusted separately within each outcome. Baseline comparisons, assumption checks, manipulation checks, sensitivity analyses, and exploratory trend analyses were excluded from the multiplicity count. Both unadjusted and Holm-adjusted *p*-values were reported. Because this multiplicity framework was formalized during revision, the adjusted secondary and exploratory findings should be interpreted as supportive rather than as results of a prospectively prespecified confirmatory testing strategy.

In the revised manuscript, the baseline-adjusted ANCOVA of post-intervention 30 m sprint time was treated as the main analysis of the prospectively registered primary outcome. The ANCOVA model was not prospectively prespecified, and the repeated-measures analysis used in the original manuscript was retained as a supportive sensitivity analysis. Because no formal a priori sample-size calculation was performed, the trial was not considered a fully confirmatory efficacy study. Analyses of secondary outcomes were interpreted as supportive, whereas technical and tactical outcomes were exploratory and hypothesis-generating. All randomized participants were analyzed in their assigned groups. Complete data were available for all outcomes analyzed in the present manuscript; therefore, no imputation was required for these analyses. All statistical tests were two-sided, with statistical significance accepted at *p* < 0.05 after the applicable multiplicity adjustment.

## 3. Results

**Figure 1:**
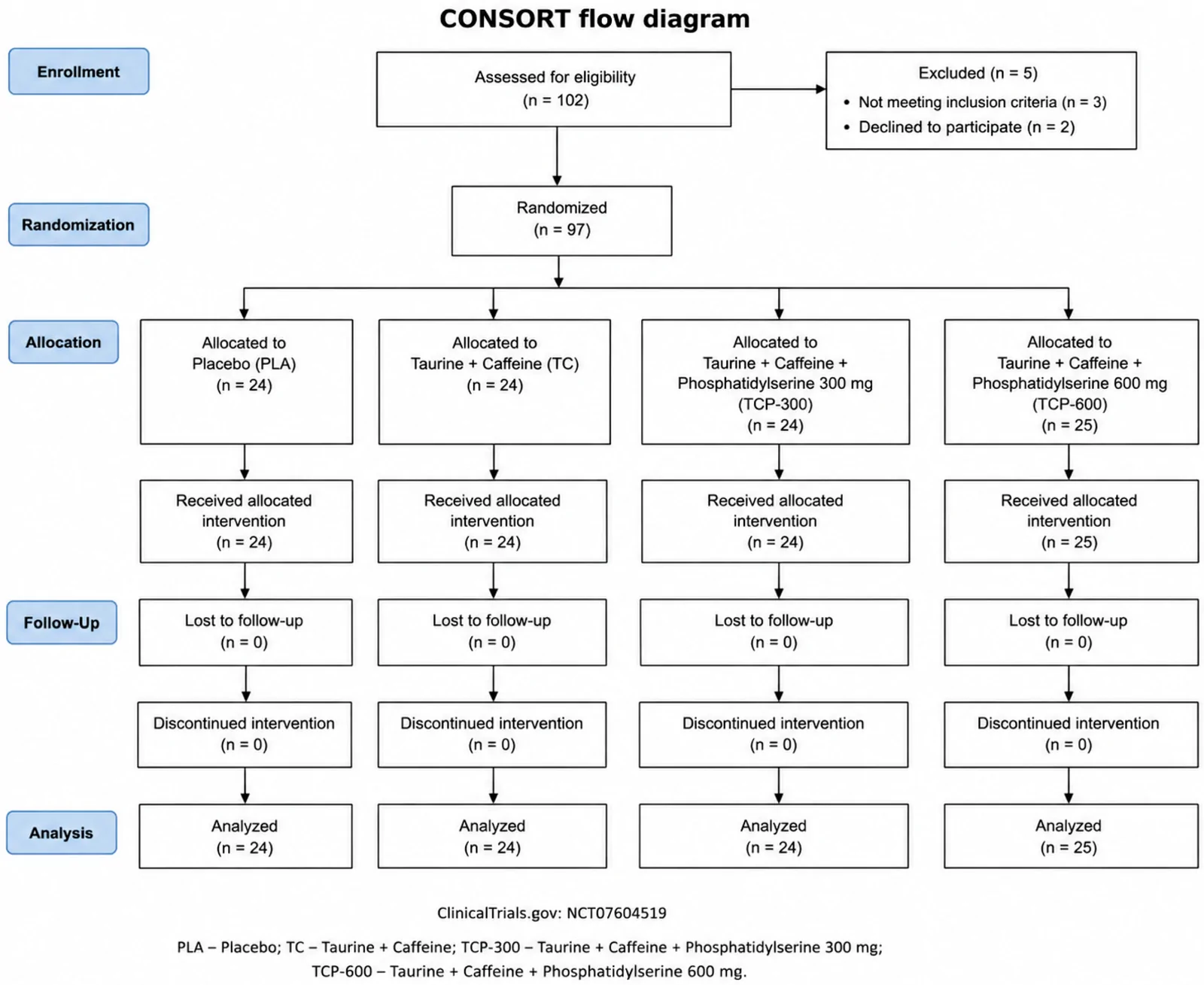
CONSORT flow diagram of participant recruitment, randomization, allocation, follow-up, and analysis. A total of 102 professional male football players were assessed for eligibility, of whom 97 met the inclusion criteria and were randomized to placebo (PLA), taurine + caffeine (TC), taurine + caffeine + phosphatidylserine 300 mg (TCP-300), or taurine + caffeine + phosphatidylserine 600 mg (TCP-600). All randomized participants completed the intervention and were included in the final analyses.

Ninety-seven players were randomized and completed the intervention. No adverse events, injuries, or protocol deviations were reported during the study period. Baseline characteristics did not differ significantly between groups. Selected Holm–Bonferroni-adjusted pairwise comparisons for outcomes that remained significant after family-level multiplicity correction are presented in Supplementary Table S1. The flow of participants throughout the study is presented in Figure 1.

### 3.1. Baseline Characteristics

Baseline anthropometric and training-related characteristics are presented in Table 1. Baseline GPS-derived locomotor variables are presented in Supplementary Table S2. Baseline psychomotor and cognitive values are shown in the baseline columns of Table 2 and Table 3. No statistically significant between-group differences were observed for age, body mass, height, body fat percentage, training experience, sprint performance, visual reaction time, auditory reaction time, Stroop-task performance, perceived mental fatigue, habitual caffeine intake, or pre-testing training load (all *p* > 0.05), indicating no evident baseline imbalance between groups. The distributions of participants by club affiliation, squad status, and primary playing position are presented in Table 1, Panel B. No significant between-group differences were observed for club affiliation (χ^2^(6) = 1.54, *p* = 0.957), squad status (Monte Carlo *p* = 0.889), or primary playing position (Monte Carlo *p* = 0.954).

Baseline GPS-derived locomotor variables assessed during the familiarization football-specific session were also comparable between groups, with no significant differences observed for total distance covered, relative distance, high-speed running distance, sprint count, maximum speed, or PlayerLoad (all *p* > 0.05; see Supplementary Table S2).

### 3.2. Cognitive Fatigue and Psychomotor Outcomes

**Figure 2:**
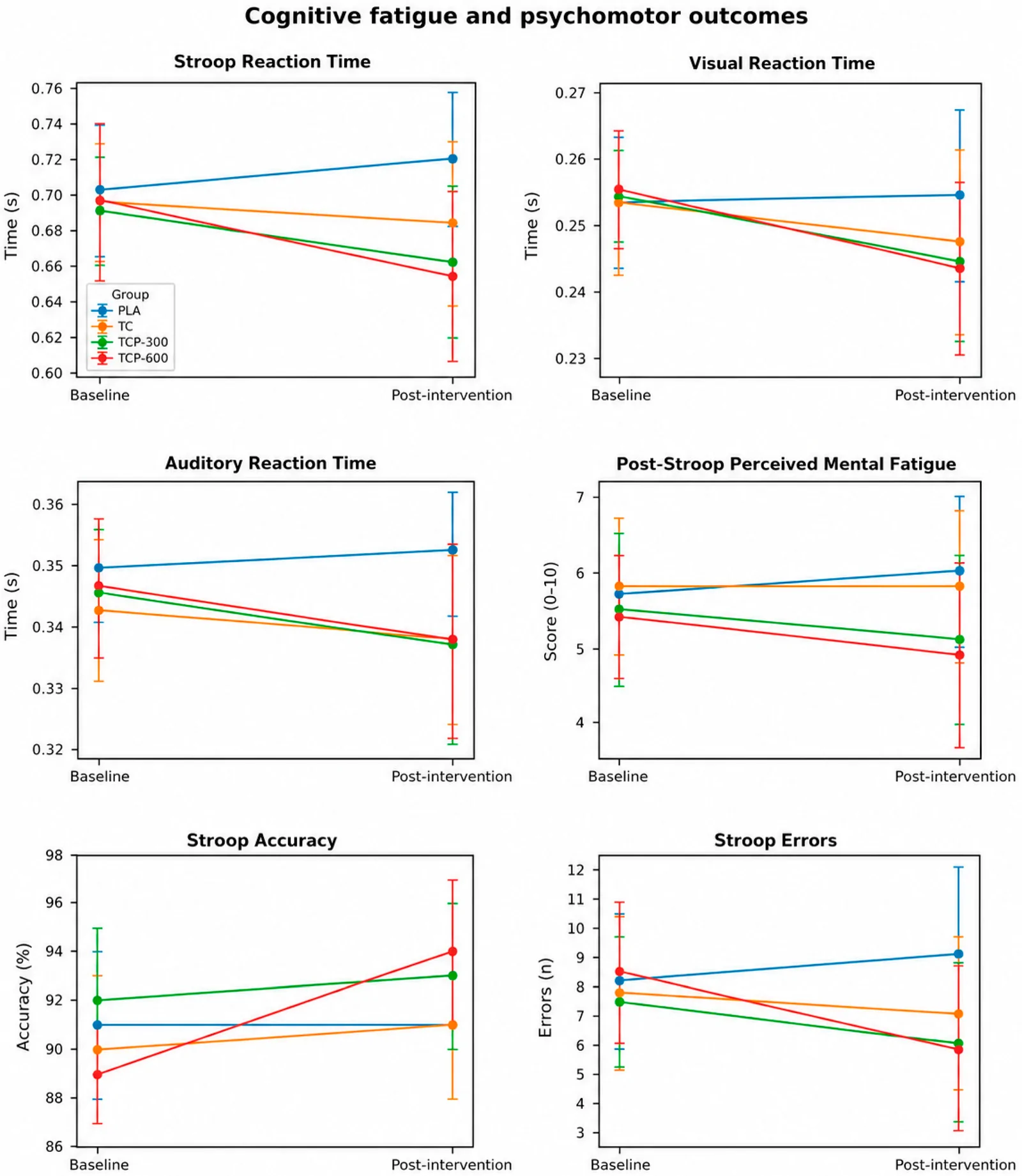
Changes in cognitive and psychomotor outcomes from baseline to post-intervention. Values are presented as mean ± standard deviation. For Stroop reaction time, visual reaction time, auditory reaction time, Stroop accuracy, and Stroop errors, treatment-related effects were evaluated using the group × intervention-time interaction from mixed-model repeated-measures ANOVA. For perceived mental fatigue, the relevant treatment-effect test was the group × intervention time × task phase interaction; the post-Stroop values displayed in the figure are descriptive. Holm–Bonferroni-adjusted *p*-values are presented in Supplementary Table S3. PLA, placebo; TC, taurine plus caffeine; TCP-300, taurine plus caffeine plus phosphatidylserine 300 mg·day^−1^; TCP-600, taurine plus caffeine plus phosphatidylserine 600 mg·day^−1^; RT, reaction time.

Changes in cognitive fatigue and psychomotor outcomes are presented in Table 2 and Figure 2.

After Holm–Bonferroni correction within the cognitive and psychomotor outcome family, significant treatment-related effects remained for perceived mental fatigue, Stroop reaction time, and visual reaction time (Holm-adjusted *p* = 0.0156, 0.0156, and 0.024, respectively). The nominal effects observed for Stroop accuracy and Stroop errors did not remain statistically significant after correction, and auditory reaction time was also non-significant (Holm-adjusted *p* = 0.096 for each outcome). At baseline, all groups demonstrated comparable Stroop-task and psychomotor performance. Following the 14-day supplementation period, the largest numerical changes were generally observed in the TCP-600 group, whereas TCP-300 also showed favorable numerical changes across several cognitive and psychomotor outcomes.

Stroop reaction time demonstrated the greatest reduction in TCP-600, followed by TCP-300 and TC. Numerically, Stroop-task accuracy increased, and Stroop error count decreased in the phosphatidylserine-containing multi-ingredient conditions; however, these outcomes did not remain statistically significant after family-level Holm–Bonferroni correction.

Only small changes were observed in PLA.

The main effect of task phase was significant—F(1, 93) = 185.32, *p* < 0.001, and ηp^2^ = 0.666 (95% CI: 0.552–0.738)—confirming an acute increase in perceived mental fatigue following the 15 min Stroop task. At baseline, mean task-induced changes ranged from 1.6 to 1.9 points across groups. At post-intervention, the task-induced increase was 1.9 points in PLA, 1.8 points in TC, 1.1 points in TCP-300, and 0.7 points in TCP-600. The group × intervention time × task phase interaction was significant, F(3, 93) = 5.10, *p* = 0.0026, ηp^2^ = 0.141 (95% CI: 0.021–0.253), indicating that the task-induced fatigue response differed between groups over the intervention period. Visual reaction time improved to a greater extent in TCP-300 and TCP-600 compared with PLA, with the largest improvement observed in TCP-600. Auditory reaction time did not show a statistically significant group × intervention-time interaction (unadjusted *p* = 0.074; Holm-adjusted *p* = 0.096; ηp^2^ = 0.071, 95% CI: 0.000–0.165).

Post hoc pairwise comparisons adjusted using the Holm–Bonferroni procedure demonstrated the most consistent differences between PLA and TCP-600.

### 3.3. Sprint Performance

**Figure 3:**
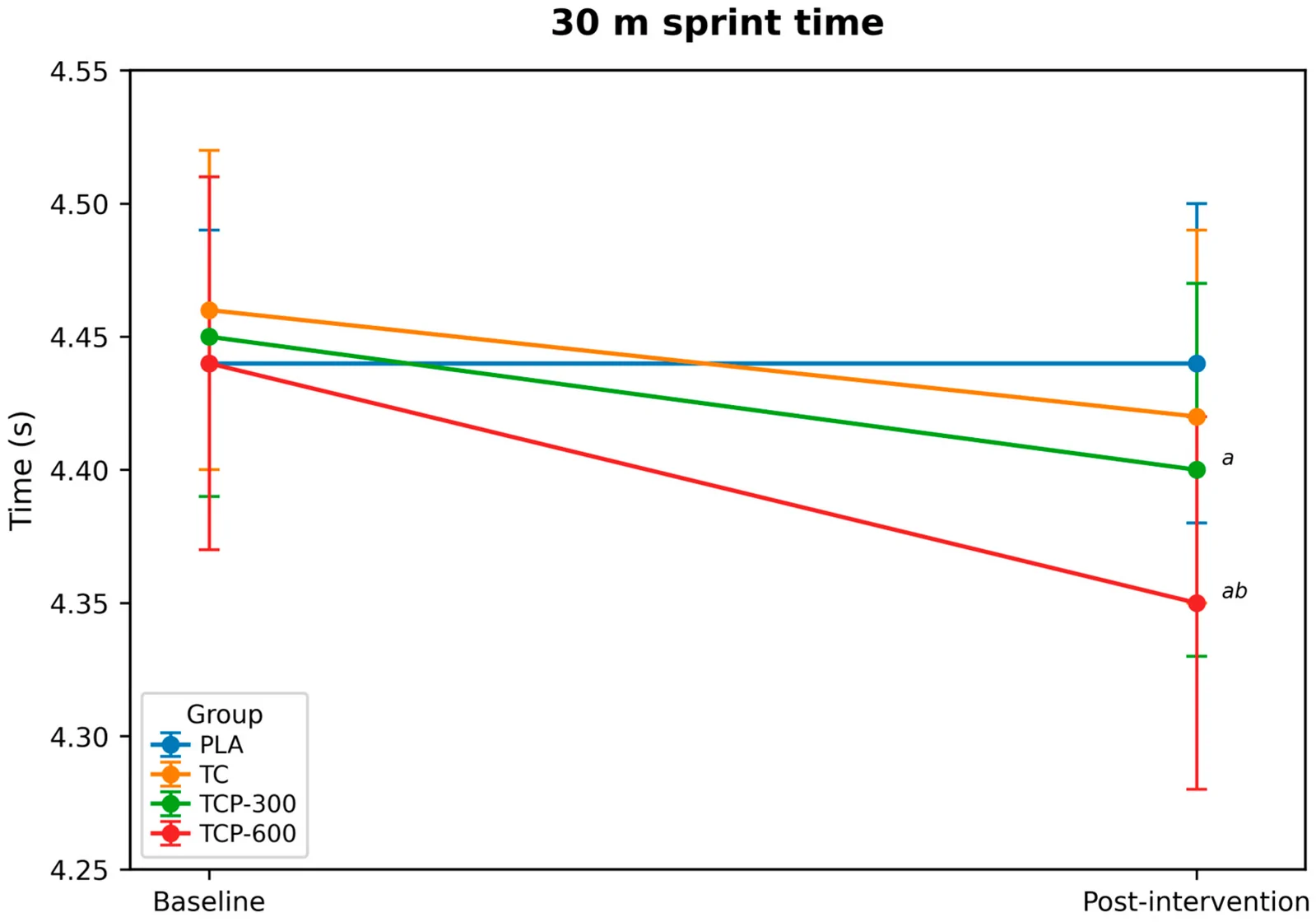
Changes in 30 m sprint time from baseline to post-intervention. Points represent means, and error bars represent standard deviations. The baseline-adjusted ANCOVA was used as the main analysis of the prospectively registered 30 m sprint outcome, whereas the group × intervention-time interaction from the mixed-model repeated-measures ANOVA was retained as a supportive sensitivity analysis. The ANCOVA model was adopted during manuscript revision and was not prospectively prespecified. Superscript ᵃ indicates a significant baseline-adjusted difference versus PLA, and superscript ᵇ indicates a significant baseline-adjusted difference versus TC, based on Holm–Bonferroni-adjusted pairwise comparisons following ANCOVA. PLA, placebo; TC, taurine plus caffeine; TCP-300, taurine plus caffeine plus phosphatidylserine 300 mg·day^−1^; TCP-600, taurine plus caffeine plus phosphatidylserine 600 mg·day^−1^.

Changes in 30 m sprint time are presented in Table 3 and Figure 3. The baseline-adjusted ANCOVA was used as the main analysis of the prospectively registered 30 m sprint outcome in the revised manuscript, whereas the repeated-measures group × intervention-time analysis was retained as a supportive sensitivity analysis. As described in the Methods, the ANCOVA model was adopted during revision and was not prospectively prespecified.

The baseline-adjusted ANCOVA demonstrated a significant overall treatment-group effect on post-intervention 30 m sprint time, F(3, 92) = 8.44, *p* = 0.000052, ηp^2^ = 0.216 (95% CI: 0.068–0.334). The supportive mixed-model repeated-measures analysis also demonstrated a significant group × time interaction (*p* = 0.002; ηp^2^ = 0.146, 95% CI: 0.024–0.259).

Baseline sprint times were comparable across groups. The largest numerical reduction in 30 m sprint time was observed in TCP-600, followed by TCP-300 and TC, whereas PLA showed minimal change. Baseline-adjusted post-intervention means were 4.444 s (95% CI: 4.421–4.467) for PLA, 4.418 s (95% CI: 4.395–4.441) for TC, 4.394 s (95% CI: 4.371–4.417) for TCP-300, and 4.364 s (95% CI: 4.341–4.387) for TCP-600.

Holm–Bonferroni-adjusted comparisons demonstrated faster adjusted sprint times in TCP-300 than in PLA (adjusted mean difference: 0.050 s, 95% CI: 0.017–0.083; *p* = 0.014), in TCP-600 than in PLA (0.080 s, 95% CI: 0.047–0.113; *p* = 0.000035), and in TCP-600 than in TC (0.054 s, 95% CI: 0.021–0.087; *p* = 0.008). The remaining comparisons were not statistically significant, including TCP-300 versus TCP-600 (0.030 s, 95% CI: −0.003–0.063; *p* = 0.227). Thus, TCP-600 showed the most favorable numerical response, although the adjusted difference between the two phosphatidylserine-containing multi-ingredient conditions was not statistically significant.

### 3.4. GPS-Derived Locomotor Variables

Baseline GPS-derived locomotor variables were comparable between groups and are presented in Supplementary Table S2. After adjustment for the corresponding baseline GPS-derived value, significant overall treatment-group effects were observed for total distance, relative distance, high-speed running distance, sprint count, maximum speed, and PlayerLoad (all unadjusted *p* ≤ 0.008012; ηp^2^ = 0.120–0.220). All six outcomes remained statistically significant after Holm–Bonferroni correction within the GPS-derived locomotor outcome family (Holm-adjusted *p* = 0.000246–0.008012).

Baseline-adjusted post-intervention estimated marginal means with 95% confidence intervals are presented in Table 4.

During the standardized football-specific exercise protocol, adjusted GPS-derived locomotor outcomes were generally more favorable in the active supplementation groups than in placebo. The most favorable values were typically observed in TCP-600, although maximum speed showed a plateau between TCP-300 and TCP-600. The largest adjusted effects were observed for high-speed running distance (ηp^2^ = 0.220) and PlayerLoad (ηp^2^ = 0.210). These findings represent baseline-adjusted post-intervention between-group differences during the standardized football-specific exercise protocol and should not be interpreted as direct evidence of pre–post improvement in locomotor performance.

### 3.5. Post-Intervention Within-Session Performance-Loss Variables

Post-intervention first-half and second-half values and the corresponding within-session decline indices are presented in Table 5.

Within-session performance-loss variables were assessed only during the post-intervention football-specific protocol and therefore represent between-group differences in first-to-second-half performance loss rather than pre–post changes. Significant between-group differences were observed for sprint decline, maximum-speed decline, high-speed-running decline, and PlayerLoad decline (exact *p* = 0.0002–0.022; ηp^2^ = 0.099–0.191). All four within-session performance-loss outcomes remained statistically significant after Holm–Bonferroni correction within the fatigue-related decline outcome family (Holm-adjusted *p* = 0.0008–0.028).

PLA demonstrated the largest negative changes from the first to the second half of the protocol, whereas TCP-600 demonstrated the smallest decline values. Sprint decline and high-speed running decline were progressively smaller across the TC, TCP-300, and TCP-600 conditions. Maximum-speed decline and PlayerLoad decline were also smaller in the phosphatidylserine-containing multi-ingredient conditions than in PLA. Inspection of the raw first-half and second-half values indicated that the lower percentage decline in TCP-300 and TCP-600 descriptively reflected both higher first-half outputs for several locomotor variables and better maintenance of output during the second half of the protocol. These findings describe differences between groups during the post-intervention session and should not be interpreted as improvements from baseline.

### 3.6. Exploratory Technical and Tactical Outcomes

Exploratory technical and tactical outcomes are presented in Table 6.

One-way ANOVA demonstrated significant omnibus between-group effects for passing accuracy, decision-making score, ball recoveries, and technical errors. All four effects remained statistically significant after Holm–Bonferroni correction within the exploratory technical and tactical outcome family (Holm-adjusted *p* = 0.000302–0.020981). However, because these observational outcomes were assessed during a single standardized football-specific session and are strongly dependent on the tactical and situational context, they should be interpreted as exploratory and hypothesis-generating rather than as confirmatory evidence of intervention efficacy. During the standardized football-specific session, the most favorable exploratory technical and tactical values were generally observed in TCP-600, whereas PLA showed the least favorable values. Passing accuracy and decision-making scores were generally more favorable across the active supplementation groups, while technical errors were lower in the phosphatidylserine-containing multi-ingredient conditions. Ball recoveries were also higher in TCP-300 and TCP-600 than in PLA.

Inter-rater reliability was good to excellent across the exploratory technical and tactical outcomes: passing accuracy, ICC = 0.91 (95% CI: 0.86–0.94); decision-making score, ICC = 0.82 (95% CI: 0.74–0.88); ball recoveries, ICC = 0.86 (95% CI: 0.79–0.91); and technical errors, ICC = 0.88 (95% CI: 0.82–0.92). Because these outcomes were exploratory and based on observational analysis performed during a single standardized football-specific session, the findings should be interpreted cautiously and regarded as hypothesis-generating rather than confirmatory evidence.

## 4. Discussion

The baseline-adjusted ANCOVA of the prospectively registered 30 m sprint outcome demonstrated a significant treatment-group effect on post-intervention sprint time. This model was adopted as the main analysis during manuscript revision, whereas the repeated-measures analysis used in the original manuscript was retained as a supportive sensitivity analysis. Compared with PLA, baseline-adjusted sprint time was faster in TCP-300 by 0.050 s (95% CI: 0.017–0.083) and in TCP-600 by 0.080 s (95% CI: 0.047–0.113). TCP-600 also showed a faster adjusted sprint time than TC by 0.054 s (95% CI: 0.021–0.087), whereas the adjusted difference between TCP-300 and TCP-600 was not statistically significant. Cognitive, psychomotor, and GPS-derived locomotor outcomes were supportive secondary findings, whereas the decline indices were interpreted separately as post-intervention between-group differences in first-to-second-half performance loss. Across the supportive secondary outcomes, numerical differences were observed among the active supplementation conditions; however, no consistent statistically significant advantage of TCP-600 over TCP-300 was demonstrated. Post hoc exploratory linear trend analyses did not support a dose–response relationship. Because phosphatidylserine was not tested independently, these findings should be interpreted as responses to the combined multi-ingredient protocols rather than as evidence of an isolated phosphatidylserine effect. Because phosphatidylserine was not tested independently, these findings should be interpreted as responses to the combined multi-ingredient protocols rather than as evidence of an isolated phosphatidylserine effect. Modern football places substantial demands on both physical and cognitive systems. Players must repeatedly perform high-intensity actions while maintaining attention, decision-making, technical execution, and tactical awareness. Previous research has shown that cognitive fatigue can impair technical skills, physical output, and decision-making quality during football-specific activity [23]. Therefore, nutritional strategies capable of attenuating both physical and cognitive fatigue may be particularly valuable in elite football.

Supportive secondary analyses indicated more favorable responses in selected cognitive and psychomotor outcomes during the standardized cognitive-load challenge. After family-level Holm–Bonferroni correction, significant treatment-related effects remained for Stroop reaction time, visual reaction time, and perceived mental fatigue. Nominal improvements were also observed for Stroop accuracy and errors, but these outcomes did not remain statistically significant after correction. Auditory reaction time showed a favorable numerical pattern but was not statistically significant after correction. These findings are consistent with a more favorable response to the brief standardized cognitive-load challenge but should not be interpreted as definitive evidence of a generalized cognitive-fatigue-resistance effect. The most favorable responses were generally observed in TCP-600. These findings are consistent with previous evidence showing that caffeine may enhance both cognitive and physical performance under fatigue-inducing conditions [24]. Improvements in high-intensity exercise performance have also been reported in team-sport athletes following caffeine supplementation [25], and even relatively low caffeine doses may provide meaningful ergogenic benefits during repeated high-intensity exercise and cognitively demanding tasks [26]. Furthermore, Raeder et al. demonstrated significant interactions between cognitive, neuromuscular, and metabolic responses during football-specific multidirectional sprint activity, highlighting the importance of maintaining cognitive function under fatigue [27].

Although more favorable numerical outcomes were generally observed in the phosphatidylserine-containing conditions than in TC, the absence of a phosphatidylserine-only group precludes attribution of these differences specifically to phosphatidylserine. The observed responses may reflect the combined effects or interactions of taurine, caffeine, and phosphatidylserine within the multi-ingredient protocols. Potential phosphatidylserine-related mechanisms involving neuronal membrane function, cognitive processing, and stress regulation remain biologically plausible [17], but these mechanisms were not directly assessed and cannot be isolated from the present design. Previous studies have reported changes in exercise capacity and physiological or endocrine responses following phosphatidylserine supplementation [28,29]. In the present trial, TCP-600 showed the most favorable numerical values across several cognitive, psychomotor, locomotor, and fatigue-related outcomes, although differences between TCP-300 and TCP-600 were modest for several measures. Because the dose comparisons and linear trend analyses were post hoc and exploratory, these findings do not establish a definitive dose–response relationship and should be regarded as hypothesis-generating. The current results also build on earlier findings reported by Mizera et al. [22], who demonstrated beneficial effects of combined caffeine-, taurine-, and phosphatidylserine-containing supplementation in professional football players. Across several psychomotor and locomotor outcomes, TCP-600 tended to show the most favorable numerical values; it also showed the smallest post-intervention first-to-second-half performance losses.

Sprint performance also improved following supplementation. The greatest reduction in 30 m sprint time was observed in TCP-600, followed by TCP-300 and TC, whereas placebo showed only minimal change. These findings are consistent with previous studies reporting performance benefits of caffeine supplementation in football players. Gallo-Salazar et al. observed improvements in selected physical performance variables following caffeine-containing supplementation in elite junior athletes [30], while Lara et al. suggested that responses to caffeine may be influenced by tolerance development over time [31]. Similar conclusions were reported in systematic reviews and meta-analyses showing beneficial effects of caffeine on repeated-sprint ability, high-intensity exercise performance, agility, and other football-specific outcomes [14,32].

The improvements in sprint performance may also be partly related to the physiological effects of taurine. Taurine is involved in calcium regulation, membrane stabilization, excitation–contraction coupling, antioxidant defense, and fatigue resistance [33]. Recent evidence indicates that taurine supplementation may enhance exercise tolerance and support high-intensity exercise performance through several complementary mechanisms [34]. In line with the present findings, Cheng et al. demonstrated that taurine attenuated declines in repeated-sprint performance and improved exercise tolerance following exhaustive exercise, with responses increasing across doses [35]. Studies combining taurine and caffeine have also reported improvements in reaction time and selected anaerobic performance measures [36], as well as longer time to exhaustion during football-related exercise under hypoxic conditions, although effects on repeated-sprint performance have been less consistent [37].

A major strength of the present study was the assessment of football-specific locomotor activity using GPS-derived variables collected during a standardized football-specific protocol. After adjustment for the corresponding baseline values, significant treatment-group effects were observed for total distance, relative distance, high-speed running distance, sprint count, maximum speed, and PlayerLoad. All six outcomes remained statistically significant after Holm–Bonferroni correction within the GPS-derived locomotor outcome family. The most favorable adjusted post-intervention values were generally observed in TCP-600, although adjusted maximum-speed values were similar in TCP-300 and TCP-600.

These findings indicate between-group differences in baseline-adjusted post-intervention locomotor output during the standardized protocol. They should not be interpreted as direct evidence of pre–post improvement or enhanced competitive match performance. The observed pattern warrants confirmation under repeated testing and official match-play conditions. Previous work has shown that multidirectional sprint training can improve change-of-direction speed and reactive agility in highly trained football players [38].

Particularly noteworthy were the between-group differences in fatigue-related decline variables assessed during the post-intervention football-specific protocol. PLA showed the largest reductions from the first to the second half, whereas TCP-600 generally showed the smallest within-session declines. The raw first-half and second-half values suggest that this pattern reflected a combination of higher initial post-intervention output and smaller subsequent reductions, rather than maintenance alone. These findings suggest that the groups differed in the magnitude of performance loss during the post-intervention session. However, because corresponding decline indices were not assessed at baseline, the results cannot be interpreted as evidence of pre–post improvement in fatigue resistance.

Accordingly, the smaller decline values observed in the phosphatidylserine-containing multi-ingredient conditions should be regarded as post-intervention between-group differences. The effect sizes for these indices quantify post-intervention between-group differences in first-to-second-half performance loss. They do not estimate an intervention-related change in fatigue resistance because comparable decline indices were not available at baseline.

This finding is especially relevant in football, where the ability to repeatedly perform high-intensity actions under fatigue is critical for performance. Recent systematic reviews have shown that repeated-sprint exercise induces substantial physiological, neuromuscular, perceptual, and performance impairments that accumulate over time [39]. Likewise, increased repeated-sprint training loads have been associated with reductions in locomotor output and neuromuscular performance [40]. Additional support comes from studies showing that caffeine-containing interventions may attenuate fatigue development and help maintain football-specific performance under demanding conditions [41]. These findings are consistent with the smaller reductions in sprint performance, high-speed running distance, and PlayerLoad observed in the present study.

At the post-intervention assessment, smaller first-to-second-half performance losses co-occurred with more favorable cognitive and perceived-fatigue outcomes in TCP-600. Previous studies have shown that cognitive fatigue may impair technical performance, reaction time, physical output, and exercise tolerance [23]. However, in the present study these outcomes were assessed concurrently, and no mediation or mechanistic analysis was performed. Therefore, no causal relationship between the cognitive and locomotor findings can be inferred, and their co-occurrence should be regarded as hypothesis-generating. Exploratory technical and tactical outcomes showed numerically favorable patterns, particularly in TCP-600. Although all four omnibus between-group effects remained statistically significant after family-level Holm–Bonferroni correction, these outcomes were exploratory, observational, and highly context-dependent because they were assessed during a single standardized football-specific session. Accordingly, they should be interpreted as hypothesis-generating rather than as confirmatory evidence of intervention efficacy. This cautious interpretation is consistent with previous evidence linking executive functioning to football-specific performance and player success [4]. Confirmation in future studies using repeated assessments and competitive match-play conditions is required. From an applied perspective, the present findings provide preliminary evidence supporting further investigation of taurine-, caffeine-, and phosphatidylserine-containing multi-ingredient protocols in professional football players. However, they should not yet be interpreted as a basis for routine supplementation recommendations in professional football. Replication in independent samples, longer intervention and follow-up periods, and validation under official match-play conditions are required before practical recommendations can be formulated. This cautious interpretation is consistent with contemporary football nutrition guidance emphasizing individualized and context-specific nutritional strategies [42]. Although taurine may theoretically complement caffeine through mechanisms related to exercise tolerance and fatigue-related pathways [34], the present design did not permit the contribution of any individual component to be isolated. The observed responses may have involved several complementary mechanisms. Caffeine-related effects on alertness and central nervous system activation [24], taurine-related effects on neuromuscular function and fatigue-related pathways [33], and phosphatidylserine-related effects on cognitive function and stress regulation [17] are biologically plausible. However, these mechanisms were not directly assessed, and the multi-ingredient design did not permit the independent contribution of any individual component to be determined. The study also has several limitations. First, the intervention period was relatively short, lasting only 14 days, and the findings should not be extrapolated to longer supplementation periods without further evidence. Biochemical, neurophysiological, and endocrine markers were not assessed, limiting mechanistic interpretation. Consequently, explanations involving central fatigue, stress-response regulation, neurotransmission, neuronal membrane function, or phosphatidylserine-related neurocognitive pathways remain speculative. The present findings therefore cannot establish the biological mechanisms underlying the observed responses. Future studies should incorporate relevant endocrine, metabolic, and neurophysiological measures to evaluate these mechanisms directly. The 15 min modified Stroop protocol has previously been used in professional soccer players [43] but has not been fully validated as a stand-alone mental-fatigue induction procedure in this population. Its relatively short duration may have elicited a milder cognitive-load response than the longer Stroop protocols commonly used in mental-fatigue research. In addition, because all active conditions contained caffeine and taurine, the independent contribution of phosphatidylserine cannot be fully isolated. Caffeine was administered as a fixed 200 mg dose rather than relative to body mass, resulting in some interindividual variation in relative caffeine exposure. Because body mass was similar across the active groups, this was unlikely to systematically confound between-group comparisons, but it may have contributed to variability in individual responses. In addition, baseline testing was performed after a seven-day period of caffeine restriction, whereas participants in all active supplementation conditions subsequently received 200 mg·day^−1^ of caffeine. Consequently, part of the observed changes in alertness, perceived mental fatigue, and psychomotor outcomes may have reflected caffeine re-exposure following short-term restriction. Because caffeine exposure was common to TC, TCP-300, and TCP-600, this factor is unlikely to explain differences among the active conditions, but it may have contributed to contrasts between the active supplementation groups and PLA. The findings for these outcomes should therefore be interpreted as responses to the complete multi-ingredient protocols under the study-specific caffeine-control conditions. Although the football-specific protocol was designed to reflect match demands, it cannot fully replicate the tactical, psychological, environmental, and competitive demands of official match play. Technical and tactical outcomes were exploratory and assessed during a single football-specific protocol; therefore, these findings should be interpreted cautiously and regarded as hypothesis-generating. Although all participants held professional contracts, the sample included first-team, reserve-team, and U19 players. The findings should therefore be generalized primarily to professional male football players training within professional club structures rather than exclusively to established first-team players. Although club affiliation, squad status, and primary playing-position distributions were comparable across the randomized groups, randomization was not stratified by these variables, and residual confounding cannot be excluded. Club affiliation was considered in sensitivity analyses of the GPS-derived outcomes; however, playing position and squad status were not included as covariates in the main GPS models. These factors may influence locomotor demands, external load, technical involvement, and tactical behavior. In addition, the outcomes obtained during a single standardized football-specific session may have been affected by club-specific practices, assigned tactical roles, team composition, interactions among players, coaching instructions, and other session-specific contextual factors. Consequently, the GPS-derived and observational technical and tactical findings should be interpreted cautiously. Masking effectiveness was not formally assessed; although treatment allocation remained concealed and the capsules were identical in appearance and packaging, some participants or study personnel may have correctly inferred the assigned condition. The registered trial comprised distinct performance, cognitive, autonomic, sleep, and perceived-recovery outcome domains. The present manuscript is restricted to the performance, cognitive, psychomotor, GPS-derived locomotor, within-session decline, and technical and tactical outcomes. The registered recovery-related outcomes are reserved for a separate companion manuscript currently in preparation and will not be duplicated across the two reports. Finally, the sample size was determined by the availability of eligible professional players rather than by a formal a priori power calculation. Consequently, the study may have had limited power to detect smaller effects, and estimates for some secondary and exploratory outcomes may be less precise. Therefore, outcomes beyond the predefined primary endpoint should be regarded as supportive or preliminary findings that require confirmation in independently powered studies. The study was also not specifically powered to compare TCP-300 with TCP-600 or to test a phosphatidylserine dose–response relationship. Consequently, modest between-condition differences may have been estimated imprecisely, and any observed numerical differences between TCP-300 and TCP-600 should be interpreted cautiously and regarded as exploratory.Although the primary outcome was predefined and multiple comparisons were adjusted, secondary findings should be interpreted as supportive, whereas technical and tactical outcomes should be regarded as exploratory and hypothesis-generating rather than definitive.

## 5. Conclusions

Under the present 14-day experimental conditions, the multi-ingredient protocols containing taurine, caffeine, and phosphatidylserine were associated with more favorable selected cognitive and psychomotor outcomes, 30 m sprint performance, and baseline-adjusted GPS-derived locomotor outcomes than placebo. The groups also differed in post-intervention first-to-second-half performance loss during the standardized football-specific protocol. Because corresponding decline indices were not assessed at baseline, these findings do not demonstrate an intervention-related improvement in fatigue resistance. Numerically more favorable values were observed for several outcomes in the TCP-600 multi-ingredient condition; however, differences relative to TCP-300 were modest and not consistently statistically significant. For the prospectively registered 30 m sprint outcome, the baseline-adjusted ANCOVA adopted in the revised manuscript indicated a significant treatment-group effect, with the repeated-measures analysis providing consistent supportive evidence. However, the adjusted comparison between TCP-300 and TCP-600 was not statistically significant. The study was not specifically powered to compare the two phosphatidylserine-containing conditions or to establish a dose–response relationship. Cognitive, psychomotor, and GPS-derived locomotor outcomes were interpreted as supportive secondary findings.

However, differences between TCP-300 and TCP-600 were modest for several outcomes, and the adjusted comparison between these conditions was not statistically significant for the predefined primary outcome. The study was not specifically powered to compare the two phosphatidylserine-containing conditions or to establish a dose–response relationship.

Technical and tactical outcomes were exploratory, observational, and hypothesis-generating and should not be interpreted as confirmatory evidence. Because phosphatidylserine was not tested independently, the findings apply to the combined taurine-, caffeine-, and phosphatidylserine-containing protocols rather than to phosphatidylserine alone. The results therefore do not establish an independent phosphatidylserine effect or a definitive advantage of the 600 mg·day^−1^ condition. Replication in independently powered trials, longer follow-up, prespecified dose comparisons, and validation under competitive match-play conditions are required before practical supplementation recommendations can be formulated.

## Figures and Tables

**Table 1:** Baseline characteristics and distribution of participants by randomized group. Continuous variables are presented as mean ± standard deviation, and categorical variables as *n* (% within group). All participants were highly trained male football players from professional football clubs. Body composition was assessed using a bioelectrical impedance analyzer (InBody 180, Biospace Co., Seoul, Republic of Korea).

**Panel A. Anthropometric and Training-Related Characteristics**					
**Variable**	**PLA (*n* = 24)**	**TC (*n* = 24)**	**TCP-300 (*n* = 24)**	**TCP-600 (*n* = 25)**	***p*** **-Value**
Age (years)	24.6 ± 3.8	24.9 ± 4.1	24.7 ± 3.7	25.0 ± 4.0	0.940
Body mass (kg)	78.8 ± 3.4	79.2 ± 3.2	78.9 ± 3.5	79.1 ± 3.3	0.860
Height (cm)	179.1 ± 4.2	178.8 ± 3.9	179.3 ± 4.1	179.0 ± 4.0	0.910
Body fat (%)	12.6 ± 2.1	12.4 ± 1.9	12.5 ± 2.0	12.5 ± 2.1	0.970
Training experience (years)	12.1 ± 3.2	12.4 ± 3.5	12.2 ± 3.1	12.5 ± 3.4	0.890
Training frequency (days/week)	6	6	6	6	—
Daily training duration (h/day)	1.8 ± 0.2	1.8 ± 0.2	1.8 ± 0.2	1.8 ± 0.2	—
Habitual caffeine intake (mg/day)	96 ± 38	101 ± 41	98 ± 36	103 ± 40	0.820
Pre-testing training load, 72 h (AU)	825 ± 92	838 ± 88	831 ± 95	842 ± 90	0.880
Session-RPE before testing (AU)	310 ± 42	318 ± 45	314 ± 43	320 ± 44	0.790
**Panel B. Club Affiliation, Squad Status, and Primary Playing Position**					
**Characteristic**	**PLA (*n* = 24)**	**TC (*n* = 24)**	**TCP-300 (*n* = 24)**	**TCP-600 (*n* = 25)**	***p*** **-Value**
**Club affiliation**					**0.957**
Club A	9 (37.5%)	7 (29.2%)	8 (33.3%)	10 (40.0%)	—
Club B	8 (33.3%)	10 (41.7%)	7 (29.2%)	8 (32.0%)	—
Club C	7 (29.2%)	7 (29.2%)	9 (37.5%)	7 (28.0%)	—
**Squad status**					**0.889**
First team	14 (58.3%)	16 (66.7%)	15 (62.5%)	17 (68.0%)	—
Reserve team	6 (25.0%)	6 (25.0%)	7 (29.2%)	4 (16.0%)	—
U19	4 (16.7%)	2 (8.3%)	2 (8.3%)	4 (16.0%)	—
**Primary playing position**					**0.954**
Goalkeeper	2 (8.3%)	3 (12.5%)	2 (8.3%)	3 (12.0%)	—
Defender	7 (29.2%)	9 (37.5%)	8 (33.3%)	8 (32.0%)	—
Midfielder	9 (37.5%)	7 (29.2%)	6 (25.0%)	10 (40.0%)	—
Forward	6 (25.0%)	5 (20.8%)	8 (33.3%)	4 (16.0%)	—

Values in Panel A are presented as mean ± standard deviation, whereas values in Panel B are presented as *n* (% within randomized group). Between-group differences in continuous variables were assessed using one-way ANOVA. Differences in categorical distributions were assessed using Pearson’s chi-square test for club affiliation and the Fisher–Freeman–Halton exact test with Monte Carlo estimation for squad status and primary playing position because of sparse expected cell counts. The reported *p*-values refer to the overall distribution within each characteristic. Training frequency and daily training duration reflected the standardized team training schedule and were therefore not subjected to between-group statistical testing. PLA, placebo; TC, taurine plus caffeine; TCP-300, taurine plus caffeine plus phosphatidylserine 300 mg·day^−1^; TCP-600, taurine plus caffeine plus phosphatidylserine 600 mg·day^−1^; AU, arbitrary units.

**Table 2:** Cognitive fatigue and psychomotor outcomes before and after the 14-day supplementation period and perceived mental fatigue responses to the Stroop task.

**Panel A. Baseline and Post-Intervention Outcomes and Group × Intervention-Time Effects**										
**Variable**	**PLA Baseline**	**PLA Post-Intervention**	**TC Baseline**	**TC Post-Intervention**	**TCP-300 Baseline**	**TCP-300 Post-Intervention**	**TCP-600 Baseline**	**TCP-600 Post-Intervention**	**Group × Time Unadjusted *p*-Value**	**ηp^2^ (95% CI)**
Stroop RT (s)	0.703 ± 0.038	0.721 ± 0.039	0.696 ± 0.034	0.684 ± 0.047	0.691 ± 0.031	0.662 ± 0.043	0.697 ± 0.045	0.654 ± 0.048	0.003	0.138 (0.019–0.250)
Visual RT (s)	0.254 ± 0.010	0.255 ± 0.013	0.254 ± 0.011	0.248 ± 0.014	0.255 ± 0.007	0.245 ± 0.012	0.256 ± 0.009	0.244 ± 0.013	0.006	0.125 (0.012–0.234)
Auditory RT (s)	0.350 ± 0.009	0.353 ± 0.011	0.343 ± 0.012	0.338 ± 0.014	0.346 ± 0.011	0.337 ± 0.016	0.347 ± 0.012	0.338 ± 0.016	0.074	0.071 (0.000–0.165)
Post-Stroop mental fatigue (0–10)	5.7 ± 0.8	6.0 ± 1.0	5.8 ± 0.9	5.8 ± 1.0	5.5 ± 1.0	5.1 ± 1.1	5.4 ± 0.8	4.9 ± 1.2	See Panel B	See Panel B
Stroop accuracy (%)	91 ± 3	91 ± 3	90 ± 3	91 ± 3	92 ± 3	93 ± 3	89 ± 2	94 ± 3	0.032	0.090 (0.000–0.190)
Stroop errors (n)	8.2 ± 2.3	9.1 ± 3.0	7.8 ± 2.6	7.1 ± 2.6	7.5 ± 2.2	6.1 ± 2.7	8.5 ± 2.4	5.9 ± 2.8	0.047	0.082 (0.000–0.179)
**Panel B. Perceived Mental Fatigue Before and After the Stroop Task**										
**Group**	**Baseline** **Pre-Task**	**Baseline** **Post-Task**		**Baseline** **Change**		**Post-Intervention** **Pre-Task**	**Post-Intervention** **Post-Task**		**Post-Intervention** **Change**	
PLA	3.8 ± 0.8	5.7 ± 0.8		1.9 ± 0.7		4.1 ± 0.9	6.0 ± 1.0		1.9 ± 0.8	
TC	4.1 ± 0.7	5.8 ± 0.9		1.7 ± 0.8		4.0 ± 0.8	5.8 ± 1.0		1.8 ± 0.7	
TCP-300	3.9 ± 0.9	5.5 ± 1.0		1.6 ± 0.9		4.0 ± 0.9	5.1 ± 1.1		1.1 ± 0.9	
TCP-600	3.6 ± 0.8	5.4 ± 0.8		1.8 ± 0.7		4.2 ± 0.9	4.9 ± 1.2		0.7 ± 1.0	
**Inferential Analysis**										
**Effect**				**F (df1, df2)**			**Exact *p*-Value**		**ηp^2^ (95% CI)**	
Task phase				185.32 (1, 93)			7.34 × 10^−24^		0.666 (0.552–0.738)	
Group × intervention time × task phase				5.10 (3, 93)			0.002597		0.141 (0.021–0.253)	

Abbreviations: PLA, placebo; TC, taurine + caffeine; TCP-300, taurine + caffeine + phosphatidylserine (300 mg); TCP-600, taurine + caffeine + phosphatidylserine (600 mg); RT, reaction time; ηp^2^, partial eta-squared. Note. Values in Panel A are presented as mean ± standard deviation. Unadjusted *p*-values and partial eta-squared effect sizes with 95% confidence intervals refer to the group × time interaction obtained from mixed-model repeated-measures ANOVA. For perceived mental fatigue, the group × intervention time × task phase interaction reported in Panel B was treated as the outcome-level treatment-effect test; the post-task values in Panel A were descriptive and were not treated as a separate efficacy test. Holm–Bonferroni-adjusted *p*-values for the cognitive and psychomotor outcome family are presented in Supplementary Table S3.

**Table 3:** Changes in 30 m sprint time before and after the 14-day supplementation period and baseline-adjusted analysis of post-intervention sprint performance.

**Panel A. Supportive Pre–Post Analysis Using Mixed-Model Repeated-Measures ANOVA**					
**Group**	**n**	**Pre, Mean ± SD (s)**	**Post, Mean ± SD (s)**	**Group × Time *p*-Value**	**ηp^2^ (95% CI)**
PLA	24	4.44 ± 0.05	4.44 ± 0.06	0.002	0.146 (0.024–0.259)
TC	24	4.46 ± 0.06	4.42 ± 0.07		
TCP-300	24	4.45 ± 0.06	4.40 ± 0.07		
TCP-600	25	4.44 ± 0.07	4.35 ± 0.07		
**Panel B. Baseline-Adjusted ANCOVA of Post-Intervention 30 m Sprint Time**					
**Group**	**Adjusted Mean (95% CI), s**		**Overall Group Effect**	**Exact *p*-Value**	**ηp^2^ (95% CI)**
PLA	4.444 (4.421–4.467)		F(3, 92) = 8.44	0.000052	0.216 (0.068–0.334)
TC	4.418 (4.395–4.441)				
TCP-300	4.394 (4.371–4.417) ᵃ				
TCP-600	4.364 (4.341–4.387) ᵃᵇ				
**Panel C. Baseline-Adjusted Pairwise Comparisons**					
**Comparison**			**Baseline-Adjusted Mean Difference, s (95% CI)**		**Holm-Adjusted *p*-Value**
PLA vs. TC			0.026 (−0.007 to 0.059)		0.245
PLA vs. TCP-300			0.050 (0.017 to 0.083)		0.014
PLA vs. TCP-600			0.080 (0.047 to 0.113)		0.000035
TC vs. TCP-300			0.024 (−0.009 to 0.057)		0.245
TC vs. TCP-600			0.054 (0.021 to 0.087)		0.008
TCP-300 vs. TCP-600			0.030 (−0.003 to 0.063)		0.227

Note. Panel A values are presented as mean ± standard deviation. The group × intervention-time *p*-value and partial eta-squared were obtained from the supportive mixed-model repeated-measures ANOVA. Panel B presents estimated marginal means with 95% confidence intervals from the baseline-adjusted ANCOVA adopted during manuscript revision as the main analysis of the prospectively registered primary outcome. The ANCOVA model itself was not prospectively prespecified. Post-intervention 30 m sprint time was entered as the dependent variable, treatment group as the fixed factor, and baseline 30 m sprint time as the covariate. Panel C presents baseline-adjusted between-group differences with 95% confidence intervals and Holm–Bonferroni-adjusted *p*-values. In Panel B, superscript letters indicate significant baseline-adjusted pairwise differences: ᵃ vs. PLA and ᵇ vs. TC. PLA, placebo; TC, taurine plus caffeine; TCP-300, taurine plus caffeine plus phosphatidylserine 300 mg·day^−1^; TCP-600, taurine plus caffeine plus phosphatidylserine 600 mg·day^−1^; CI, confidence interval; ηp^2^, partial eta-squared.

**Table 4:** Baseline-adjusted post-intervention GPS-derived locomotor outcomes during the standardized football-specific exercise protocol.

Variable	PLA Post-Intervention Adjusted Mean (95% CI)	TC Post-Intervention Adjusted Mean (95% CI)	TCP-300 Post-Intervention Adjusted Mean (95% CI)	TCP-600 Post-Intervention Adjusted Mean (95% CI)	Unadjusted *p*-Value	ηp^2^ (95% CI)
Total distance (km)	7.06 (6.89–7.23)	7.27 (7.10–7.44)	7.46 (7.29–7.63)	7.57 (7.40–7.74)	0.000372	0.180 (0.043–0.297)
Relative distance (m·min^−1^)	78.4 (76.4–80.4)	80.8 (78.8–82.8)	82.9 (80.9–84.9)	83.9 (81.9–85.9)	0.001068	0.160 (0.031–0.275)
High-speed running distance (m)	689 (644–734)	728 (683–773)	806 (761–851)	832 (788–876)	0.000041	0.220 (0.071–0.339)
Sprint count (n)	18.1 (15.8–20.4)	21.0 (18.7–23.3)	23.5 (21.2–25.8)	23.9 (21.6–26.2)	0.001790	0.150 (0.025–0.264)
Maximum speed (km·h^−1^)	29.86 (29.34–30.38)	30.40 (29.88–30.92)	31.00 (30.48–31.52)	30.94 (30.43–31.45)	0.008012	0.120 (0.010–0.228)
PlayerLoad (AU)	784 (757–811)	824 (797–851)	861 (834–888)	869 (842–896)	0.000072	0.210 (0.064–0.328)

Note. Values are baseline-adjusted post-intervention estimated marginal means with 95% confidence intervals obtained from separate ANCOVA models. For each GPS-derived outcome, the post-intervention value was entered as the dependent variable, treatment group as the fixed factor, and the corresponding baseline value of the same outcome as the covariate. Thus, the values presented are not raw post-intervention means. Unadjusted *p*-values and partial eta-squared values refer to the overall baseline-adjusted treatment-group effect. Holm–Bonferroni-adjusted *p*-values are presented in Supplementary Table S3. Partial eta-squared values are presented with two-sided 95% confidence intervals derived using the noncentral F distribution. PLA, placebo; TC, taurine plus caffeine; TCP-300, taurine plus caffeine plus phosphatidylserine 300 mg·day^−1^; TCP-600, taurine plus caffeine plus phosphatidylserine 600 mg·day^−1^; AU, arbitrary units; CI, confidence interval; ηp^2^, partial eta-squared.

**Table 5:** Post-intervention first-half and second-half locomotor values and within-session percentage decline during the standardized football-specific exercise protocol.

Outcome	Group	First Half, Mean ± SD	Second Half, Mean ± SD	Decline, Mean ± SD (%)	Unadjusted *p*-Value	ηp^2^ (95% CI)
**Sprint count (n)**	PLA	10.9 ± 2.3	7.2 ± 1.8	−33.3 ± 5.0	0.001	0.161 (0.031–0.276)
	TC	12.1 ± 2.4	8.9 ± 2.0	−26.5 ± 5.4		
	TCP-300	13.2 ± 2.7	10.3 ± 2.3	−21.8 ± 5.1		
	TCP-600	13.2 ± 2.8	10.7 ± 2.4	−19.2 ± 5.0		
**Maximum speed** **(km·h^−1^)**	PLA	29.86 ± 1.00	25.38 ± 1.25	−15.0 ± 3.0	0.014	0.108 (0.005–0.214)
	TC	30.40 ± 1.10	26.51 ± 1.30	−12.8 ± 3.2		
	TCP-300	31.00 ± 1.10	27.78 ± 1.35	−10.4 ± 3.1		
	TCP-600	30.94 ± 1.20	27.81 ± 1.42	−10.1 ± 3.0		
**High-speed running distance (m)**	PLA	412 ± 70	277 ± 57	−32.9 ± 5.5	0.0002	0.191 (0.051–0.308)
	TC	423 ± 75	305 ± 62	−27.8 ± 5.3		
	TCP-300	455 ± 79	351 ± 70	−23.0 ± 5.1		
	TCP-600	462 ± 82	370 ± 73	−20.1 ± 5.2		
**PlayerLoad (AU)**	PLA	428.4 ± 37.0	355.6 ± 34.0	−17.0 ± 3.0	0.022	0.099 (0.001–0.202)
	TC	442.5 ± 39.0	381.5 ± 35.0	−13.8 ± 3.3		
	TCP-300	456.8 ± 43.0	404.2 ± 39.0	−11.5 ± 3.0		
	TCP-600	459.8 ± 47.0	409.2 ± 43.0	−11.0 ± 3.2		

Note. First-half and second-half values are presented as mean ± standard deviation and refer to the post-intervention standardized football-specific exercise protocol. Within-session decline was calculated separately for each participant as [(second-half value − first-half value)/first-half value] × 100, with negative values indicating a reduction from the first to the second half. Corresponding decline indices were not assessed at baseline; therefore, these results represent post-intervention between-group differences in within-session performance decline and should not be interpreted as pre–post improvements. Unadjusted *p*-values and partial eta-squared values were obtained from ANCOVA models comparing the decline indices between groups, with baseline 30 m sprint time included as the adjustment covariate. Holm–Bonferroni-adjusted *p*-values are presented in Supplementary Table S3. Partial eta-squared values are presented with two-sided 95% confidence intervals derived using the noncentral F distribution. PLA, placebo; TC, taurine plus caffeine; TCP-300, taurine plus caffeine plus phosphatidylserine 300 mg·day^−1^; TCP-600, taurine plus caffeine plus phosphatidylserine 600 mg·day^−1^; HSR, high-speed running; AU, arbitrary units; ηp^2^, partial eta-squared.

**Table 6:** Exploratory technical and tactical outcomes recorded during the standardized football-specific exercise protocol. These outcomes were exploratory and were not designated as primary or confirmatory outcomes.

Variable	PLA	TC	TCP-300	TCP-600	Unadjusted *p*-Value	Holm-Adjusted *p*-Value	ηp^2^
Passing accuracy (%)	78.2 ± 4.4	80.1 ± 4.1	81.0 ± 3.9	82.0 ± 3.7	0.011127	0.020980	0.112
Decision-making score (1–10)	6.7 ± 0.8	7.1 ± 0.7	7.3 ± 0.7	7.4 ± 0.6	0.004173	0.012519	0.132
Ball recoveries (n)	7.4 ± 1.6	8.1 ± 1.5	8.5 ± 1.5	8.8 ± 1.4	0.010490	0.020980	0.113
Technical errors (n)	8.5 ± 1.7	7.3 ± 1.6	6.9 ± 1.5	6.4 ± 1.4	0.000076	0.000304	0.207

Note. Values are presented as mean ± standard deviation. Unadjusted *p*-values were obtained from one-way ANOVA. Holm-adjusted *p*-values were calculated within the four-outcome exploratory technical and tactical family. All four omnibus effects remained statistically significant after family-level correction; nevertheless, these outcomes were not primary or confirmatory and should be interpreted as exploratory and hypothesis-generating. Partial eta-squared values refer to the omnibus between-group effect. PLA, placebo; TC, taurine plus caffeine; TCP-300, taurine plus caffeine plus phosphatidylserine 300 mg·day^−1^; TCP-600, taurine plus caffeine plus phosphatidylserine 600 mg·day^−1^; ηp^2^, partial eta-squared. Higher decision-making scores indicate more appropriate tactical decisions.

## Data Availability

The data presented in this study are available on reasonable request from the corresponding author. The data are not publicly available due to confidentiality restrictions related to the participation of professional athletes.

## Supplementary Materials

The following supporting information can be downloaded at https://www.mdpi.com/article/10.3390/nu18162588/s1, Table S1: Selected Holm–Bonferroni-adjusted pairwise comparisons for outcomes that remained significant after family-level multiplicity correction; Table S2: Baseline GPS-derived locomotor variables assessed during the pre-intervention familiarization football-specific session. Values are presented as mean ± SD. No significant between-group differences were observed (all *p* > 0.05); Table S3: Family-level Holm–Bonferroni correction of outcome-level treatment-effect tests.
