# Supplementary material for: Football-Specific Performance and Cognitive Fatigue Resistance Following Supplementation with Two Phosphatidylserine Doses Combined with Taurine and Caffeine in Professional Male Football Players: A Randomized, Triple-Masked, Placebo-Controlled Trial
Source: Nutrients. 2026 Aug 7;18(16):2588. doi: 10.3390/nu18162588 (PMC13516145; doi:10.3390/nu18162588)
Supplement: Supplementary file 1 [file nutrients-18-02588-s001.zip › nutrients-4440642-supplementary.pdf]

nutrients

MDPI

Supplementary Materials

Supplementary Table S1. Selected Holm–Bonferroni-adjusted pairwise comparisons for outcomes that remained significant after family-level multiplicity correction.

Variable	Comparison			Holm-adjusted p
Stroop reaction time	PLA vs. TCP-600			<0.001
30 m sprint time	PLA vs. TCP-600			<0.001
High-speed running distance	PLA vs. TCP-600			<0.001
Sprint decline	PLA vs. TCP-600			<0.001
Passing accuracy	PLA vs. TCP-600			0.018

Abbreviations: PLA, placebo; TCP-600, taurine + caffeine + phosphatidylserine supplementation (600 mg/day);

Note: Pairwise comparisons were interpreted only for outcomes that remained statistically significant after Holm–Bonferroni correction of the corresponding outcome family. Only selected comparisons are presented.

Supplementary Table S2. Baseline GPS-derived locomotor variables assessed during the pre-intervention familiarization football-specific session. Values are presented as mean ± SD. No significant between-group differences were observed (all p > 0.05).

Variable	PLA	TC	TCP-300	TCP-600	p
Total distance (km)	7.00 ± 0.35	7.03 ± 0.34	7.01 ± 0.36	7.04 ± 0.35	> 0.05
Relative distance ($m·min^{-1}$)	77.8 ± 3.9	78.1 ± 3.8	77.9 ± 3.8	78.2 ± 3.7	> 0.05
HSR distance (m)	705 ± 85	712 ± 83	708 ± 84	714 ± 82	> 0.05
Sprint count (n)	15.8 ± 3.0	16.0 ± 3.0	15.9 ± 2.9	16.1 ± 3.0	> 0.05
Maximum speed ($km·h^{-1}$)	30.1 ± 1.1	30.2 ± 1.0	30.2 ± 1.0	30.3 ± 1.1	> 0.05
PlayerLoad (AU)	780 ± 42	786 ± 41	783 ± 41	788 ± 40	> 0.05

Supplementary Table S3. Family-level Holm–Bonferroni correction of outcome-level treatment-effect tests.

**Outcome family**	**Outcome-level treatment-effect test**	**Unadjusted p-value**	**Holm-adjusted p-value**	**Significant after correction**
Primary	30 m sprint time, baseline-adjusted ANCOVA	0.000052	N/A	Yes
Cognitive and psychomotor	Perceived mental fatigue: group × intervention time × task phase	0.002597	0.015582	Yes
Cognitive and psychomotor	Stroop reaction time	0.003000	0.015582	Yes
Cognitive and psychomotor	Visual reaction time	0.006000	0.024000	Yes
Cognitive and psychomotor	Stroop accuracy	0.032000	0.096000	No
Cognitive and psychomotor	Stroop errors	0.047000	0.096000	No
Cognitive and psychomotor	Auditory reaction time	0.074000	0.096000	No
GPS-derived locomotor	Total distance	0.000372	0.001488	Yes
GPS-derived locomotor	Relative distance	0.001068	0.003204	Yes
GPS-derived locomotor	High-speed running distance	0.000041	0.000246	Yes
GPS-derived locomotor	Sprint count	0.001790	0.003580	Yes
GPS-derived locomotor	Maximum speed	0.008012	0.008012	Yes
GPS-derived locomotor	PlayerLoad	0.000072	0.000360	Yes
Within-session performance loss	Sprint decline	0.001000	0.003000	Yes
Within-session performance loss	Maximum-speed decline	0.014000	0.028000	Yes
Within-session performance loss	High-speed running decline	0.000200	0.000800	Yes
Within-session performance loss	PlayerLoad decline	0.022000	0.028000	Yes
Exploratory technical and tactical	Passing accuracy	0.011127	0.020980	Yes
Exploratory technical and tactical	Decision-making score	0.004173	0.012519	Yes

Outcome family	Outcome-level treatment-effect test	Unadjusted p-value	Holm-adjusted p-value	Significant after correction
Exploratory technical and tactical	Ball recoveries	0.010490	0.020980	Yes
Exploratory technical and tactical	Technical errors	0.000076	0.000304	Yes

Note. A total of 21 outcome-level treatment-effect tests were organized into five conceptually defined families. No multiplicity adjustment was applied to the prospectively registered primary outcome because it comprised a single outcome. Within each supportive secondary or exploratory family, unadjusted omnibus p-values were adjusted using the Holm–Bonferroni step-down procedure. For perceived mental fatigue, the group × intervention time × task phase interaction was treated as the single outcome-level treatment-effect test. In the revised manuscript, the baseline-adjusted ANCOVA was adopted as the main analysis of the prospectively registered 30 m sprint outcome. The mixed-model repeated-measures analysis reported in the original manuscript was retained as a supportive sensitivity analysis and was not counted as a separate outcome-level treatment-effect test. Baseline-balance analyses, model-assumption checks, manipulation checks, sensitivity analyses, and exploratory linear-trend analyses were not included in the multiplicity count. The baseline-adjusted ANCOVA model, the five-family multiplicity structure, and the primary/supportive/exploratory interpretive hierarchy were formalized during manuscript revision, after group unblinding and examination of the initial outcome results, and were therefore not prospectively prespecified. ANCOVA, analysis of covariance; N/A, not applicable.

https://doi.org/10.3390/xxxxx
